# Compton-based prompt gamma imaging using ordered origin ensemble algorithm with resolution recovery in proton therapy

**DOI:** 10.1038/s41598-018-37623-2

**Published:** 2019-02-04

**Authors:** Zhiyang Yao, Yongshun Xiao, Zhiqiang Chen, Bo Wang, Qinhan Hou

**Affiliations:** 1 Department of Engineering Physics, Tsinghua University, Beijing, 100084 China; 2 Key Laboratory of Particle & Radiation Imaging (Tsinghua University), Ministry of Education, Beijing, China

## Abstract

Prompt gamma ray (PG) imaging based on Compton camera (CC) is promising to realize *in vivo* verification during the proton therapy. However, the finite spatial and energy resolution of current CC, as well as the Doppler broaden effect, degrade the quality and resolution of PG images. In addition, due to the inherent geometrical complexity of Compton camera data, PG imaging can be time-consuming and difficult to reconstruct in real-time, while using standard techniques such as filtered back-projection or maximum likelihood-expectation maximization. In this paper, we propose three modifications of origin ensembles with resolution recovery (OE-RR) algorithm based on Markov chains to accelerate the convergence to equilibrium of OE-RR algorithm and improve the image quality. For evaluation, we performed a Monte Carlo simulation of a three-stage CZT Compton camera with resolution loss to detect the PG produced by a proton beam in a water phantom, and evaluate image quality of the gamma rays emitted during proton irradiation. The results show that our ordered OE-RR algorithm realized a good resolution recovery and accurate estimation of the position, including the peak and the distal falloff of the PG emission with remarkably faster reconstruction, thus demonstrating the feasibility of this new method in non-idealized PG-based proton range verification.

## Introduction

The proton therapy (PT) for treating cancer has been widely used over the past decades, because of the Bragg peak of proton beam. The sharp Bragg peak (BP) and the finite range of the beam provide a unique benefit for cancer radiotherapy, allowing for dose escalation to the tumor and a reduction of exposure to the surrounding healthy tissues^1^. However, the uncertainties in the position of the distal falloff restrict our ability to exploit the steep dose gradients at the distal edge of the BP, reducing the full clinical potential of proton radiation therapy^2^. Therefore, *in vivo* verification is essential to guarantee the treatment effect of PT.

PG based *in vivo* verification in proton therapy has been proved feasible for clinical application^3^. Alternatively, different approaches to develop a PG imaging (PGI) system for proton range verification are under development, including several variations of slit and pinhole cameras, time-of-flight and integral PG techniques using fast scintillators and Compton cameras (CCs). The big challenge for providing a clinically viable PGI system is related to the measure adequate signals in the energy range of PGs, emitted from tissues (2–10 MeV) during treatment delivery to ensure that the beam range within 2 mm or even less for its determination^4^.

CCs are generally composed of two detectors that are used to detect photons^5^. The photons’ scattering angle can be determined by the measured deposited energies in these detectors^6^. With the development of detectors and detection technology, CCs have attracted much more attention in *in-vivo* verification during the proton therapy due to its potential in full-space range measurement without mechanical collimation, which is promising to reduce the range uncertainty^7^. However, the image resolution that can be achieved by the real-world systems based on CC is seriously limited by the available detectors’ energy and spatial resolution, as well as the Doppler broadening, restricting the performance of the CC systems^8^. In addition to optimizing camera design, the image reconstruction algorithm, which could be geared to compensate for these resolution-limiting effects, will be essential for the future clinical applications of CC.

Currently, several methods have been proposed to solve the problem. The list-mode ordered subset expectation maximization (OSEM) with the shift-variant point spread functions (LM-OSEM-SV-PSFs) can recover the reconstructed degrade image by incorporating the SV-PSFs into the iteration process^9^. And this algorithm worked well for the reconstruction of proton-induced PGs^10^. However, the SV-PSF parameters estimation is time-consuming and have to change as the detection environment (including the detector geometry, field of view (FOV), source characteristics) changes, making the clinical real-time image reconstruction difficult to achieve. Besides, one previous study showed that the origin ensemble with resolution recovery (OE-RR) algorithm based on Markov chain^8^, which is an extended (stochastic origin ensembles) SOE algorithm^11^ including resolution recovery (RR), has good performance in terms of image quality while clearly outperforming in reconstruction time. In their research, 511 keV multiple point-like sources with four groups CC were simulated and used to demonstrate the feasibility of this algorithm in resolution correction. However, the convergence to equilibrium of OE-RR was slower than the original SOE, and the store of the coefficients’ matrix presented a challenge in the real-time reconstruction of large amounts of data such as PG. Meanwhile, as far as we know, no research has used the OE-RR algorithm to reconstruct the PGs induced by the proton beam.

In this study, PG induced by proton pencil beam were simulated and detected by a three-stage Compton camera with finite energy and spatial resolution. We assess the performance of PGs images reconstructed by OE-RR with Monte Carlo simulated data. We evaluate how well OE-RR could reproduce the peak and distal falloff of the proton beam, as well as how well it could improve the spatial resolution of reconstructions. In order to speed up the reconstruction by OE-RR and optimize the image quality, we made three modifications of OE-RR to improve its performance for PGI. Both the effects of the modifications were also evaluated. To evaluate the feasibility of range verification with non-idealized CC, a large water phantom (20 cm × 20 cm × 20 cm) similar to patients’ size were chosen. Besides, the different reconstructions of characteristic PG were also evaluated.

## Results

### Origin ensembles based reconstruction

**Figure 1:**
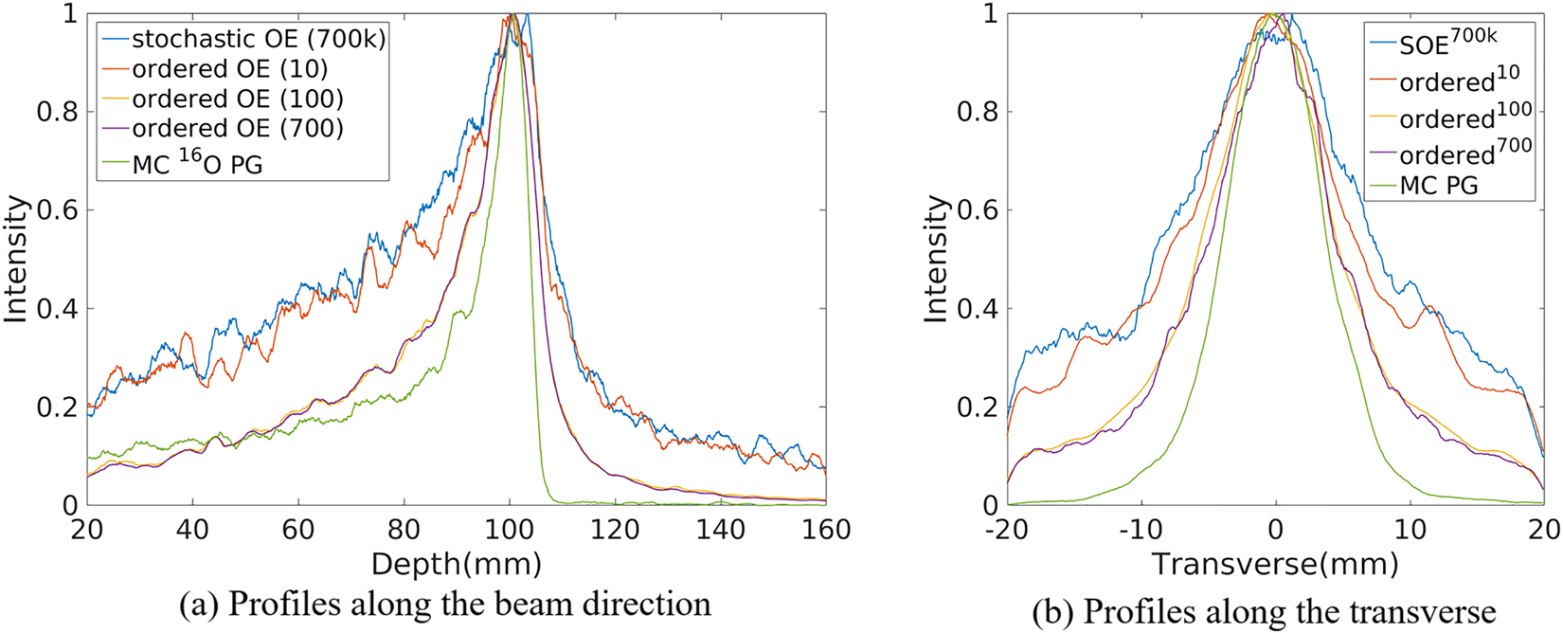
Profiles of the 2D projection ^16^O PG images reconstructed by origin ensembles (OE) algorithms (i.e. stochastic OE^11^ with 700,000 OE iterations and ordered OE^2^ with different iterations) and obtained by Monte Carlo simulation (true value), respectively.

The PG emissions were produced by a 120 MeV proton pencil beam (2D Gaussian spatial profile σ = 3 mm, total number = 1.0 × 10^10^), irradiating a large water phantom (20 cm length × 20 cm width × 20 cm depth) in our simulation. The 2D projection PG images were based on the volume of interest (VOI) 2000 × 2000 × 1 bins, the size of each bin being 0.1 mm × 0.1 mm × 0.1 mm. The center of the VOI in depth along the center of the phantom (center of the beam). They were 2D reconstruction since our reconstructions is to evaluate the difference between the reconstruction and the true value (Monte Carlo simulations) in the same spatial location (i.e. the same plane). The horizontal profiles were taken the central line of 2D projection images along the direction and perpendicular to the direction of proton beam, respectively. The horizontal profiles were created by taking the central line of the 2D image along the beam direction and transverse direction. The 2D projection images used for obtaining the horizontal profiles were followed by a 2D Gaussian post-smoothing filter with a small full-width half-maximum (FWHM) 2.0 mm to reduce the background noise and reproduce peaks better. The peak positions were obtained by recording the location of the point with a value of “1”. The 80% and 50% falloff positions are obtained by linear interpolation with a maximum scale 0.1 mm. Three repeated independent simulations were implemented to reduce random error. Figure 1 shows the profiles of 2D projection ^16^O PG images reconstructed by the SOE^11^ and OE with event ordered modification^2^. The number of events used for reconstruction in Fig. 1 is about 70,000. Thus, 10 times ordered OE iterations are equal to 700,000 stochastic OE iterations with regard to the numbers of OE iterations. For a better comparison of OE algorithm, the interaction positions and deposited energies of events were exact, regardless of detection error. The narrower width at 80% positions and better reproduction of falloff and peak positions obtained by ordered OE in Fig. 1 and Table 1, show that event ordered used for OE iterations could provide a better reconstruction compared with event stochastic used. Besides, as shown in Table 1, the modification for event ordered can accelerate the reconstruction of OE algorithm due to its simpler iterative process without randomly selecting events.

**Table 1:** The comparison of reconstruction by using stochastic OE algorithm and ordered OE algorithm with different OE iterations.

Methods		Peak (mm)	80% fall off (mm)	50% fall off (mm)	The width at depth 80%(mm)	The width at 80% transverse (mm)	Reconstr-uction time(s)
MC ^16^O PG	70,000 events	100.80 ± 0.01	102.84 ± 0.01	104.15 ± 0.01	4.20 ± 0.01	4.97 ± 0.05	—
stochastic OE	700,000 OE iterations	103.35 ± 0.14	105.29 ± 0.12	108.66 ± 0.10	9.96 ± 0.25	7.66 ± 0.07	675
ordered OE	10 ordered OE iterations	100.37 ± 0.33	105.31 ± 0.06	107.41 ± 0.08	8.70 ± 0.14	6.96 ± 0.01	2.98
ordered OE	100 ordered OE iterations	100.60 ± 0.01	103.84 ± 0.07	105.94 ± 0.01	6.97 ± 0.06	5.57 ± 0.01	10.8
ordered OE	700 ordered OE iterations	100.87 ± 0.09	102.44 ± 0.43	105.35 ± 0.85	7.11 ± 0.09	5.60 ± 0.02	65.1

Values of positions in the table represent the mean position (mm) ± standard deviation (mm) of three repeated reconstructions.

As Table 1 shows, 100 times iterations for ordered OE algorithm with 10^4^ events were sufficient to obtain the accurate estimation and high spatial resolution for PG imaging. Thus, the SOE without resolution recovery (abbreviated as SOE) and OE-RR in our study were ordered event used to accelerate the reconstruction process.

**Figure 2:**
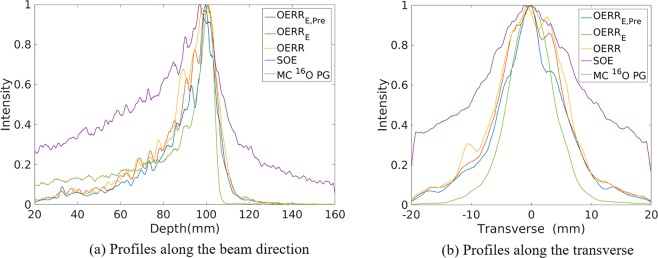
Profiles of the 2D projection ^16^O PG images reconstructed by origin ensembles (OE) algorithm and obtained by Monte Carlo simulation (true value), respectively.

**Figure 3:**
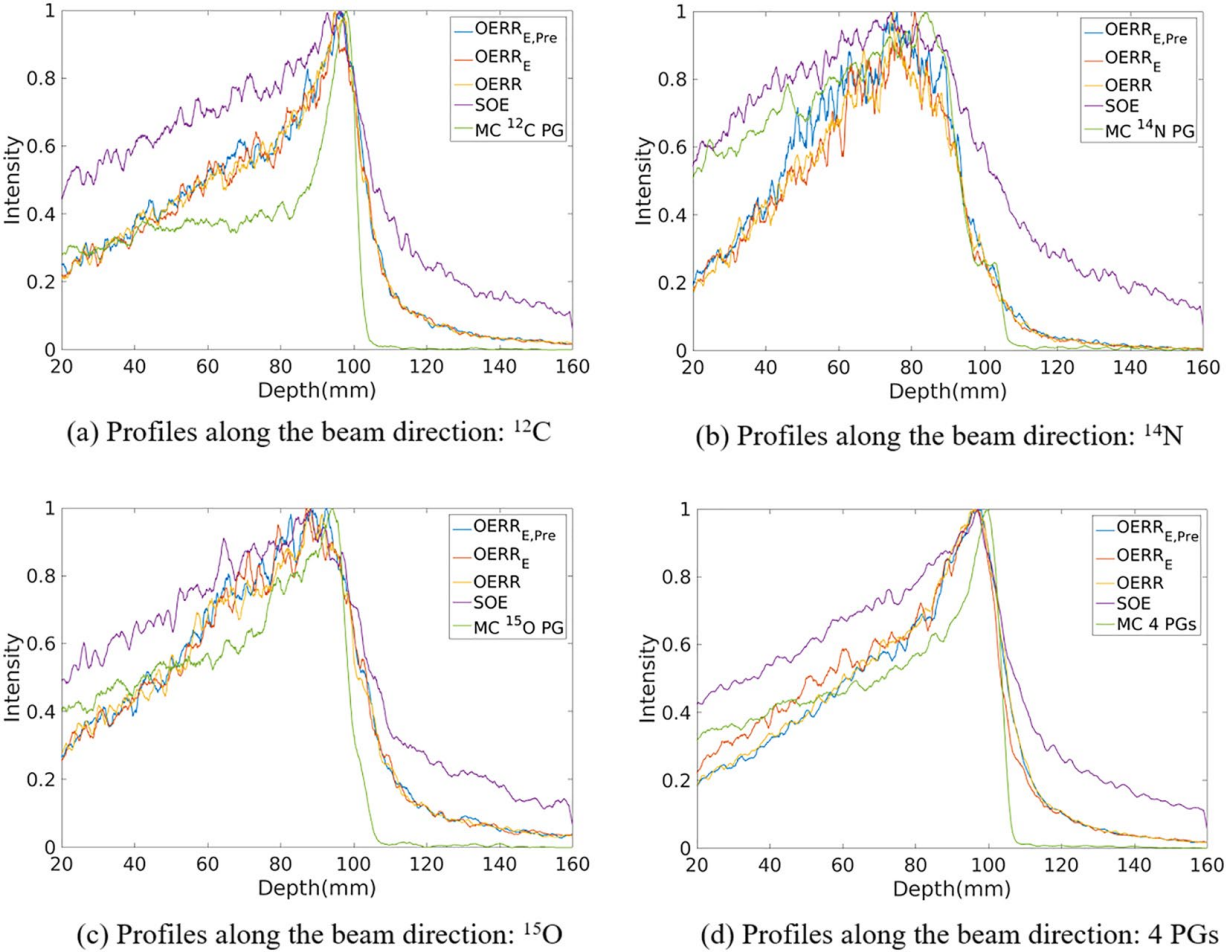
Profiles of the 2D projection PG images (^12^C, ^14^N, ^15^O, ^12^C + ^14^N + ^15^O + ^16^O, respectively) reconstructed by origin ensembles (OE) algorithm and obtained by Monte Carlo simulation (true value), respectively.

Figure 2 shows the horizontal profiles of the 2D projection ^16^O PG images reconstructed by SOE, three kinds of OE-RR, as well as obtained by Monte Carlo simulation. The three kinds of OE-RR consist of OERR with the corrections of pre-calculation and deposited energies (OERR_E,Pre_), OERR with the correction of the deposited energies (OERR_E_) and OERR without the corrections of the pre-calculation and deposited energies (OERR). The method of pre-calculation correction for OERR_E,Pre_ will be presented in section 4.2 (i.e. pre-calculation for initial states of OE-RR), and the method of deposited energies correction for OERR_E,Pre_ and OERR_E_ will be presented in section 4.3. The detection of PG used a three stage Cadmium Zinc Telluride (CZT) detector with spatial resolution of 1 mm in landscape and 1 mm in the depth, energy resolution of 1.64% for ^137^Cs (662 keV). The total coincidence events used for reconstruction was about 137000, 123000, 70000 and 75000 for ^14^N, ^12^C, ^15^O and ^16^O de-excitations, respectively. Figure 3 compares the horizontal profiles of the 2D projection different characteristic PG images obtained by origin ensembles (OE) algorithms and Monte Carlo simulation.

As shown in Figs 2 and 3, it can be observed that the FWHM or the width at 80% obtained by both three kinds of OE-RR is less than that of SOE. Besides, compared with SOE, the peak positions and fall-off distal obtained by OE-RR have a better agreement with the true value (i.e. Monte Carlo simulation). Thus, the OE-RR reconstructions provide better spatial resolution while the SOE reconstructions are distorted due to the finite spatial and energy resolution of CC. The improvement by OE-RR is shown in Tables 2 and 3, specifically.

**Table 2:** The PGs’ peak and falloff positions(mm) obtained by the Monte Carlo simulation (true), SOE, OE-RR with the corrections of pre-calculation and deposited energies (OERR_E,Pre_), OERR with the correction of the deposited energies (OERR_E_) and OERR without the corrections of the pre-calculation and deposited energies (OERR).

PG emissions		^14^N + ^12^C + ^15^O + ^16^O	^14^N	^12^C	^15^O	^16^O
Peak	TRUE	99.80 ± 0.09	83.90 ± 0.02	97.77 ± 0.22	94.67 ± 0.20	100.80 ± 0.05
	OERR_E,Pre_	97.90 ± 0.12	75.80 ± 1.27	97.23 ± 0.01	93.03 ± 0.01	100.10 ± 0.30
	OERR_E_	95.80 ± 0.10	78.93 ± 0.83	95.30 ± 0.02	90.07 ± 1.94	99.50 ± 0.07
	OERR	95.63 ± 0.48	75.80 ± 2.89	95.93 ± 0.01	90.40 ± 1.38	101.10 ± 0.44
	SOE	97.80 ± 0.19	74.23 ± 0.07	96.50 ± 0.02	88.33 ± 0.54	97.10 ± 0.05
80%	TRUE	101.95 ± 0.18	89.41 ± 1.03	99.86 ± 0.01	96.84 ± 0.02	102.48 ± 0.52
	OERR_E,Pre_	100.90 ± 0.10	89.83 ± 0.05	99.77 ± 0.34	97.21 ± 0.23	101.64 ± 0.33
	OERR_E_	100.55 ± 0.01	87.49 ± 0.49	99.88 ± 0.54	97.26 ± 0.19	104.03 ± 0.04
	OERR	100.50 ± 0.10	86.52 ± 0.63	99.63 ± 0.13	96.76 ± 0.03	103.62 ± 0.02
	SOE	100.71 ± 1.58	92.02 ± 0.21	100.23 ± 0.09	98.10 ± 1.63	103.66 ± 0.11
50%	TRUE	103.91 ± 0.11	97.03 ± 1.25	102.41 ± 0.01	98.50 ± 0.28	103.51 ± 0.82
	OERR_E,Pre_	104.96 ± 0.01	94.91 ± 1.40	103.18 ± 0.32	101.85 ± 2.37	105.80 ± 0.71
	OERR_E_	103.19 ± 0.05	94.89 ± 2.54	103.36 ± 0.14	102.13 ± 1.43	107.09 ± 0.01
	OERR	105.29 ± 0.02	94.61 ± 1.51	103.11 ± 0.26	101.73 ± 2.93	107.22 ± 0.09
	SOE	107.12 ± 0.35	104.06 ± 3.32	105.40 ± 0.18	105.27 ± 0.05	109.05 ± 0.04

Values in the table represent the mean position (mm) ± standard deviation (mm) of three repeated independent simulations.

**Table 3:** The width at 80% positions(mm) obtained by the Monte Carlo simulation (true), SOE, OE-RR with the corrections of pre-calculation and deposited energies (OERR_E,Pre_), OERR with the correction of the deposited energies (OERR_E_) and OERR without the corrections of the pre-calculation and deposited energies (OERR).

PG emissions		^14^N + ^12^C + ^15^O + ^16^O	^14^N	^12^C	^15^O	^16^O
The width at 80% along beam direction	TRUE	6.61 ± 0.76	24.26 ± 3.24	5.86 ± 0.51	6.83 ± 1.79	4.20 ± 0.66
	OERR_E,Pre_	9.89 ± 1.57	28.46 ± 2.44	9.33 ± 1.41	18.41 ± 1.70	3.59 ± 0.68
	OERR_E_	11.81 ± 1.76	21.93 ± 3.04	10.44 ± 0.56	19.52 ± 1.10	5.97 ± 1.14
	OERR	12.89 ± 1.28	28.18 ± 2.89	11.99 ± 0.36	20.28 ± 1.34	8.05 ± 1.60
	SOE	20.30 ± 2.33	47.15 ± 5.03	17.81 ± 3.98	22.81 ± 1.85	14.08 ± 2.02
The width at 80% transverse direction	TRUE	4.89 ± 0.66	3.20 ± 1.41	4.21 ± 1.41	5.12 ± 1.42	5.01 ± 1.70
	OERR_E,Pre_	7.17 ± 1.89	8.75 ± 0.94	6.94 ± 0.57	7.83 ± 1.39	3.48 ± 1.48
	OERR_E_	7.91 ± 2.35	9.80 ± 0.78	6.92 ± 0.87	9.53 ± 0.91	7.57 ± 1.44
	OERR	8.03 ± 1.49	8.63 ± 1.38	7.52 ± 1.03	9.29 ± 1.94	7.83 ± 0.83
	SOE	9.35 ± 1.34	15.48 ± 0.81	9.86 ± 0.76	12.22 ± 2.67	9.47 ± 1.38

Values in the table represent the mean (mm) ± standard deviation (mm) of three repeated independent simulations.

Moreover, even though the reconstructions obtained by three OERRs were similar, the peak positions reconstructed by OERR_E,Pre_ were generally closer to that obtained by Monte Carlo simulation such as the ^14^N + ^12^C + ^15^O + ^16^O PGs within 2 mm error in Table 2. In addition, as shown in Table 3, the width at 80% positions along the beam direction obtained by the OERR_E,Pre_ are less than the others for ^12^C, ^14^N and ^16^O PG emissions, and the width at 80% positions of transverse direction obtained by the OERR_E,Pre_ are less than the others for ^15^O and ^16^O PG emissions. Thus, OE-RR with correction of energies provided the better resolution recovery compared with the other two.

As Table 2 shows, for the large phantom simulation, both the reconstructions by OE-RR algorithms could provide accurate estimation for the 80% falloff positions of PGs distribution within 2 mm. Besides, the reconstruction by OERR_E,Pre_ could predict the peak positions of ^12^C, ^15^O, ^16^O and ^14^N + ^12^C + ^15^O + ^16^O PGs obtained by Monte Carlo simulation with an accuracy of less than 1.9 mm. Moreover, OERR_E,Pre_ based reconstructions reproduced the peak position and the falloff of ^16^O with the accuracy of less than 1.65 mm. These results demonstrate the feasibility of OERR_E,Pre_ based reconstruction for range verification with large phantom.

The results of OERR_E_ and OERR in Table 3 show that the modification of deposited energies worked well for high energy PG like ^16^O (6.13 MeV), but for lower energy such as ^14^N (2.31 MeV), the modification has little effects. Besides, by comparing the results of OERR_E,Pre_ and OERR_E_ in Tables 2 and 3, we find that the modification of pre-calculation of probability densities in VOI performed better for ^12^C, ^15^O and ^16^O since their true PG distribution were more concentrated. That is the OERR_E,Pre_ led more convergent results compared with OERR_E_. However, for ^14^N PG, whose distribution is like a line. The peak position obtained by OERR_E,Pre_ is worse than that obtained by OERR_E_. Considering that ^14^N PG distribution is in worse agreement with Bragg peak compared with ^12^C, ^15^O and ^16^O, OERR_E,Pre_ is more suitable for PGs reconstruction.

### Reconstructed PGs image

**Figure 4:**
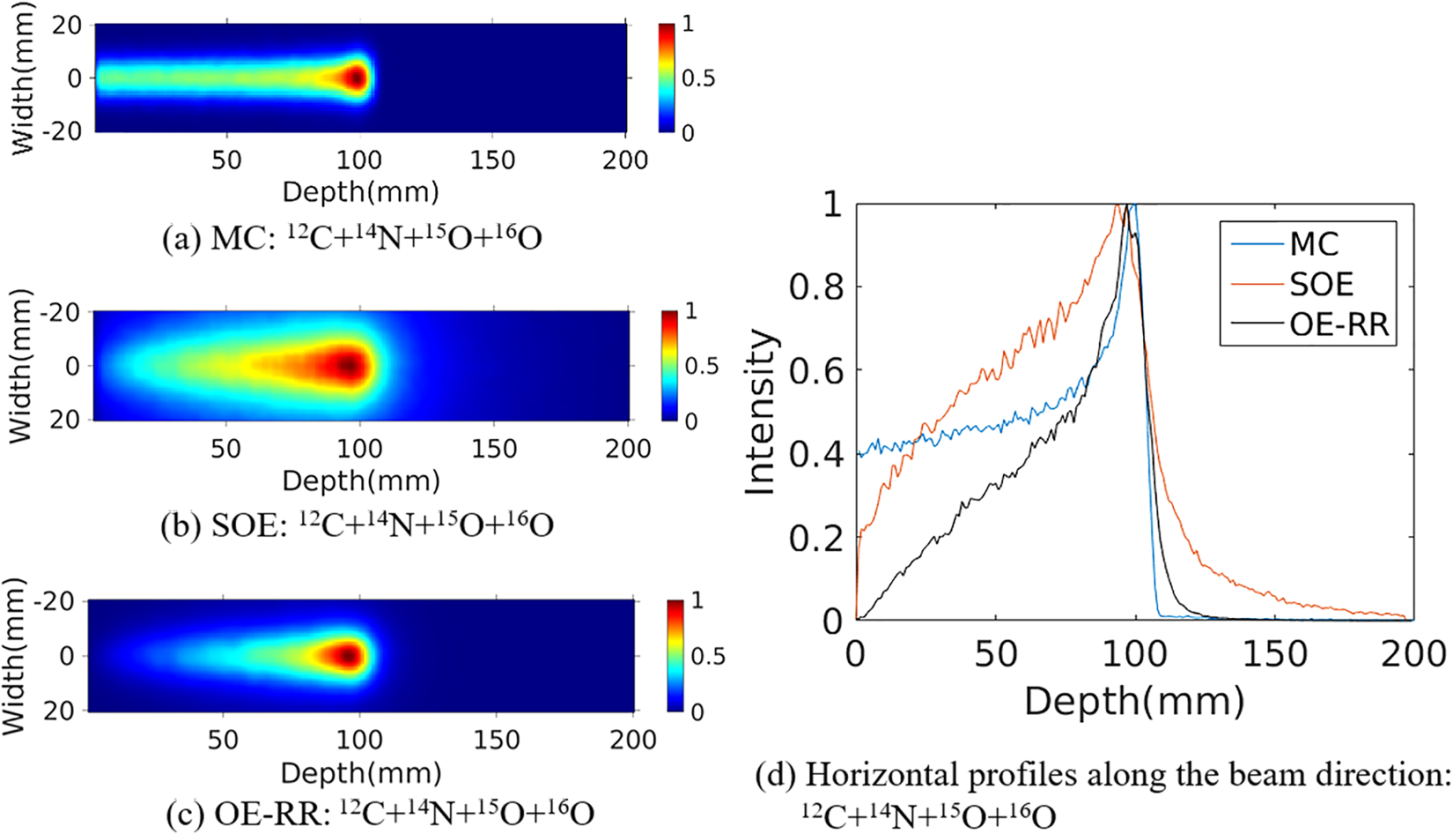
2D PG images (^14^N + ^12^C + ^15^O + ^16^O) obtained by (**a**) Monte Carlo simulation (true). (**b**) SOE without resolution recovery, (**c**) OE-RR. (**d**) Horizontal profiles along the beam direction (central of the beam) of PG images (^14^N + ^12^C + ^15^O + ^16^O) obtained by Monte Carlo simulation (true), SOE without RR and OE-RR.

**Figure 5:**
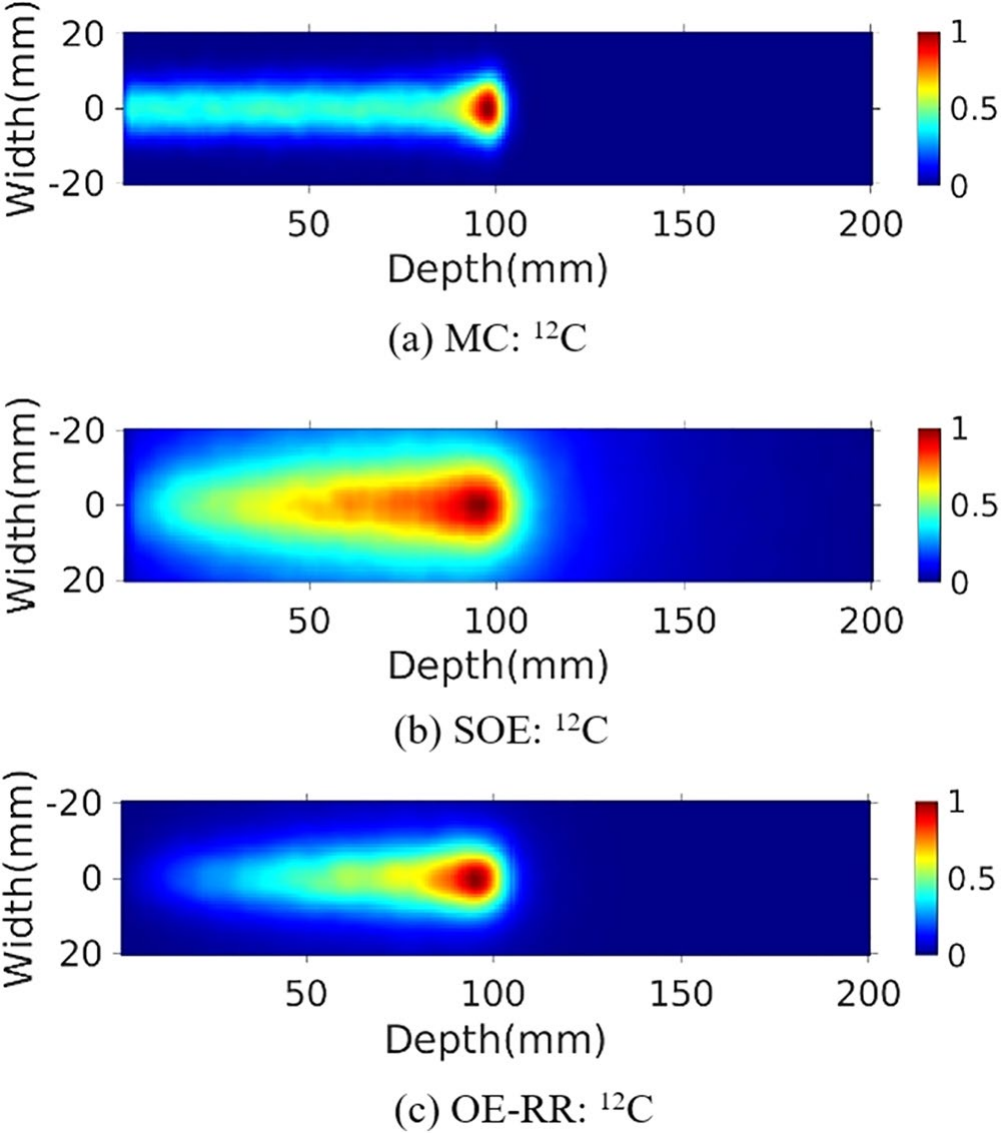
2D PG image (^12^C) obtained by (**a**) MC simulation, (**b**) SOE without resolution recovery, (**c**) OE-RR.

**Figure 6:**
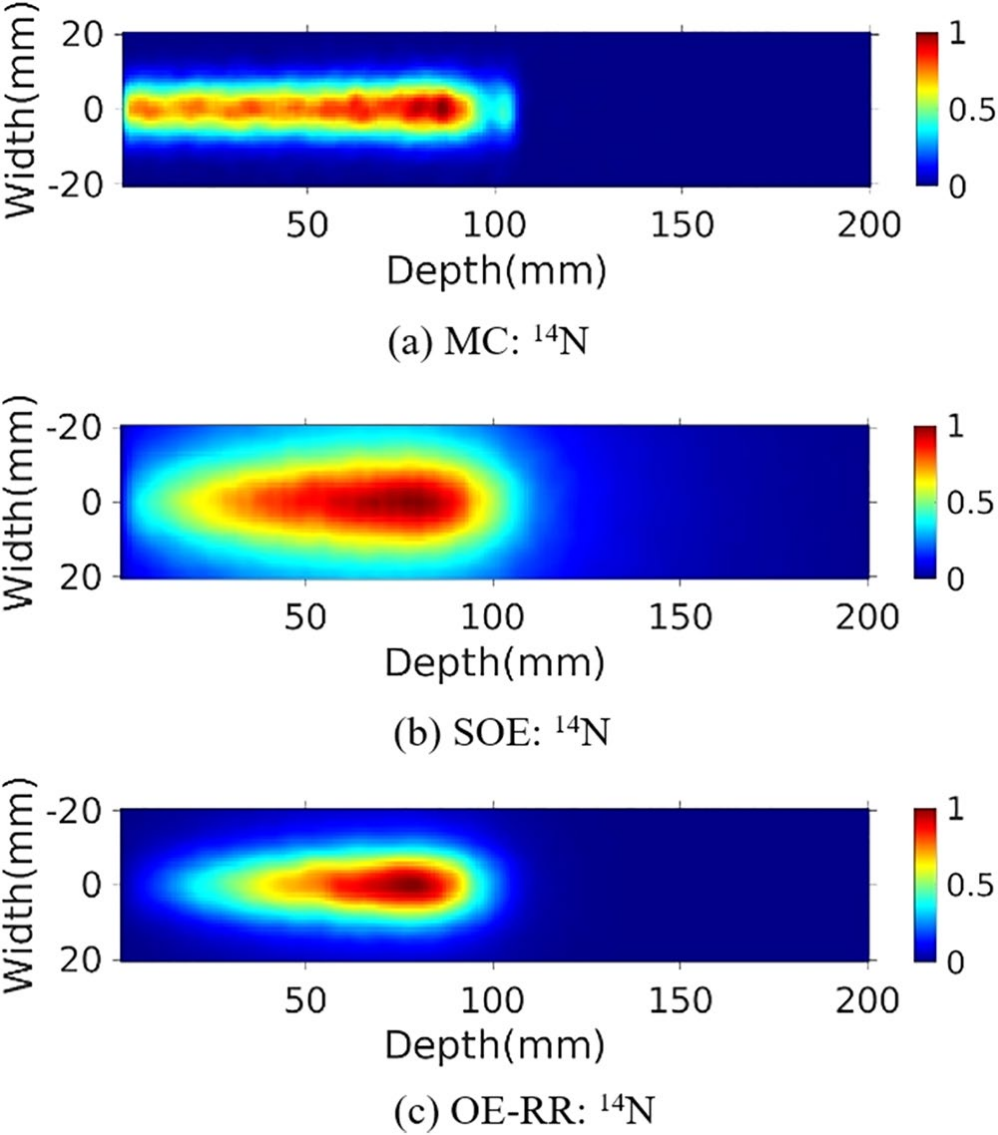
2D PG image (^14^N) obtained by (**a**) MC simulation, (**b**) SOE without resolution recovery, (**c**) OE-RR.

**Figure 7:**
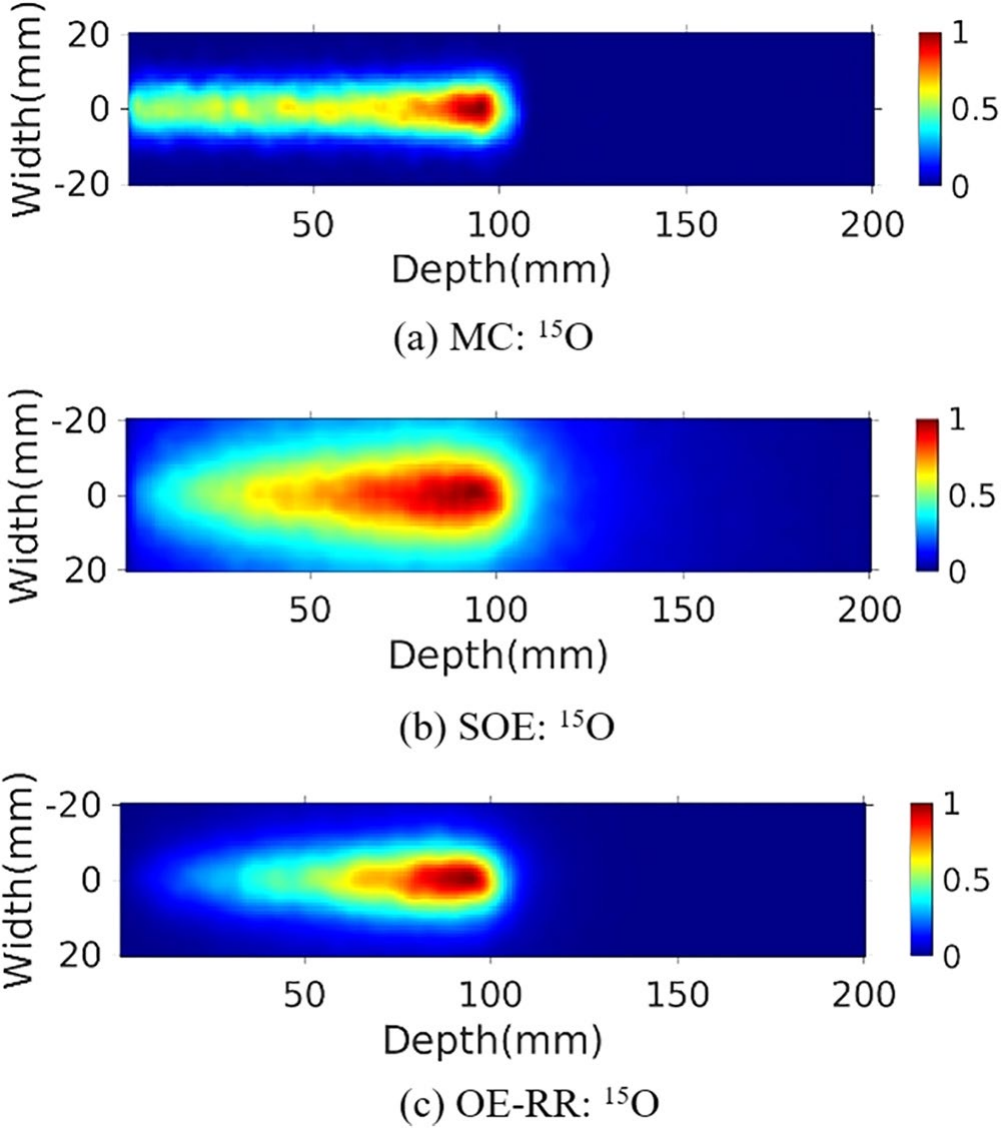
2D PG image (^15^O) obtained by (**a**) MC simulation, (**b**) SOE without resolution recovery, (**c**) OE-RR.

**Figure 8:**
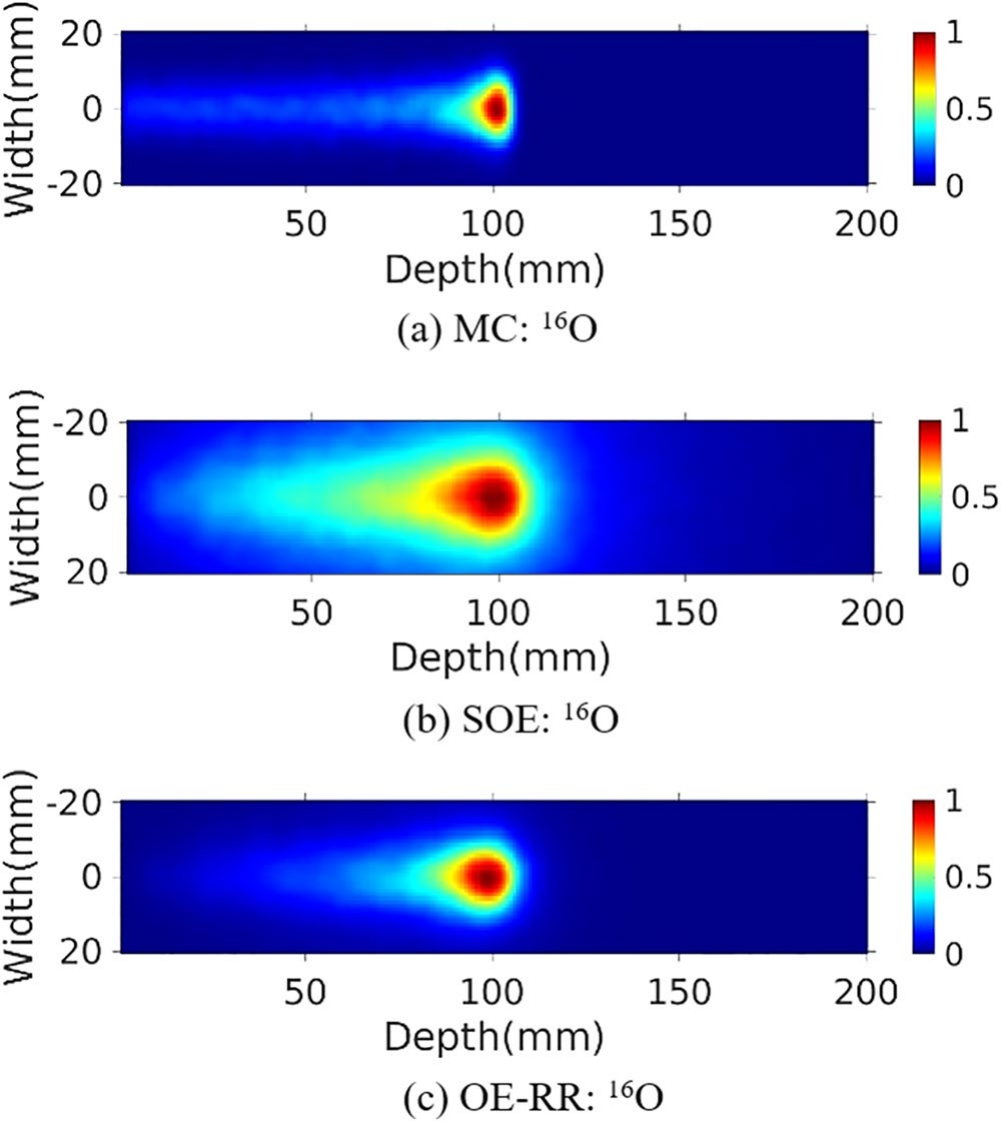
2D PG image (^16^O) obtained by (**a**) MC simulation, (**b**) SOE without resolution recovery, (**c**) OE-RR.

**Figure 9:**
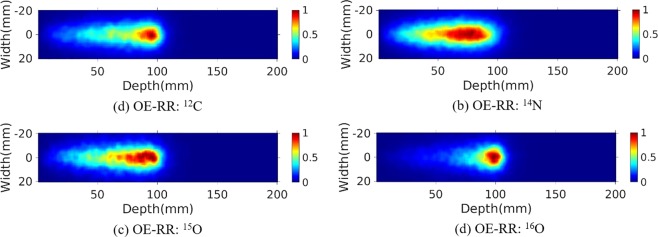
Plane along the center of the phantom (center of the beam) of 3D reconstructions for ^14^N, ^12^C, ^15^O and ^16^O PG obtained by OE-RR, respectively.

The water phantom (10 cm length × 10 cm width × 20 cm depth) were chosen for further PG measurement and evaluation of reconstructions. A 120 MeV proton pencil beam (2D Gaussian spatial profile σ = 5 mm, total number = 1 × 10^9^) was irradiated to the phantom to simulate the small area treatment in proton therapy. The three-stage CZT detectors with spatial resolution of 2 mm in landscape and 1 mm in the depth direction, energy resolution of 1.64% for ^137^Cs (662 keV) were used. The number of coincidence triple-events used for reconstruction was about 184 000, and the number of coincidence double-events was about 64 000. The total coincidence events used for reconstruction was about 88 000, 74 000, 41 000 and 45 000 for ^14^N, ^12^C, ^15^O and ^16^O de-excitations, respectively. We make a comparison of 2D projection ^14^N + ^12^C + ^15^O + ^16^O PG images obtained by Monte Carlo simulation, and reconstructed by SOE and OE-RR (Here OE-RR refers to OERR_E,Pre_) in Fig. 4. The 2D projection PG images were based on the VOI 200 × 200 × 1 bins, the size of each bin being 1 mm × 1 mm × 1 mm. In each reconstruction, more than four million OE iterations were implemented to obtain the results. Besides, reconstructed images of characteristic PG produced from ^14^N, ^12^C, ^15^O and ^16^O de-excitations were shown in Figs 5–8. It is observed that OE-RR reconstruction provided better image quality for all the PG emissions. However, the reconstruction for PG derived from ^14^N had a large peak width, limiting the improvement provided by the OE-RR. For 3D reconstructions, the VOI was chosen 200 × 200 × 80 bins, the size of each bin being 1 mm × 1 mm × 1 mm. The plane along the center of the phantom (center of the beam) of 3D reconstructions were shown in Fig. 9.

**Figure 10:**
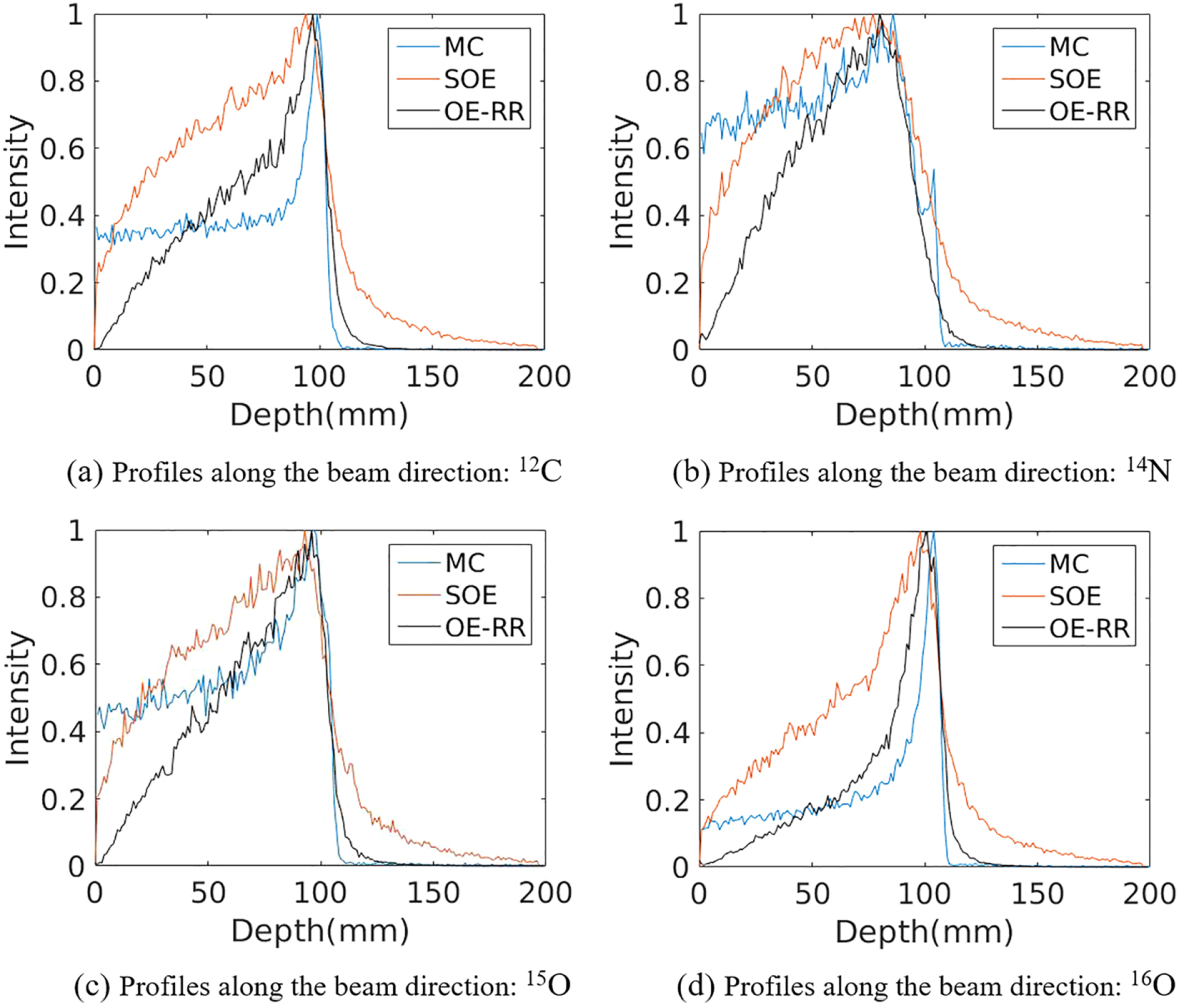
Horizontal profiles (along the center of beam) of ^14^N, ^12^C, ^15^O and ^16^O PG obtained by MC simulation (true), SOE and OE-RR, respectively.

Figure 4(d) shows horizontal profiles of ^14^N + ^12^C + ^15^O + ^16^O PGs obtained by Monte Carlo simulation, and reconstructed by SOE and OE-RR. The horizontal profiles were created by taking the central line of the 2D images along the beam direction. The 2D projection images used for obtaining the horizontal profiles were followed by a 2D Gaussian post-smoothing filter with FWHM equals 2.0 mm, to reduce noise and reproduce peak better. The peak positions were obtained by recording the location of the point with a value of “1”. The 80% and 50% falloff positions are obtained by linear interpolation with a maximum scale 1.0 mm. The horizontal profiles of the four kinds of PG images were shown in Fig. 10, respectively. The results show that the distal edges of the OE-RR reconstructions were found in good agreement with that obtained by Monte Carlo (true value). Besides, the less FWHM of OE-RR reconstruction provided a better spatial resolution compared with SOE reconstruction. However, for PG derived from ^14^N de-excitation, OE-RR could not improve the agreement between the Monte Carlo PG and reconstructed PG.

### Range verification

Table 4 shows the depths of the peak position, 80% and 50% falloff positions for ^14^N + ^12^C + ^15^O + ^16^O, ^14^N, ^12^C, ^15^O and ^16^O PG emissions obtained by Monte Carlo simulation, SOE without RR and OE-RR. Compared with the reconstruction using SOE, OE-RR-based reconstruction provided better estimates of both the peak position, 80% and 50% falloff positions. Compared with the peak position, the 80% and 50% falloff positions can be more accurately estimated for OE-based reconstruction. Our results also show that the reconstruction using OE-RR can be used to predict the 80% and 50% falloff positions of ^14^N + ^12^C + ^15^O + ^16^O, ^12^C, ^15^O and ^16^O PG emissions with an accuracy of less than 1.6 mm. For PG emission derived from ^14^N, the difference between the peak position and 50% falloff positions of reconstructed by OE-RR and that of Monte Carlo simulation was large (>6 mm). However, the OE-RR reconstruction can predict the 80% falloff positions of it to within 1.4 mm. The mean errors of the peak, 80% and 50% falloff positions(mm) of PG emissions between the Monte Carlo simulation (true) and that calculated by SOE and OE-RR are shown in Table 5.

**Table 4:** The PGs’ peak and falloff at 80% and 50% positions(mm) obtained by the Monte Carlo simulation (true), SOE without resolution recovery, OE-RR.

PG emissions		^14^N + ^12^C + ^15^O + ^16^O	^14^N	^12^C	^15^O	^16^O
Peak	TRUE	99.97 ± 0.89	86.09 ± 0.08	100.24 ± 0.66	97.53 ± 3.01	101.89 ± 0.09
	SOE	95.92 ± 3.61	79.44 ± 5.98	94.78 ± 0.39	91.78 ± 4.14	97.49 ± 1.82
	OE-RR	98.07 ± 0.90	82.56 ± 3.91	97.57 ± 0.51	95.22 ± 1.57	100.77 ± 1.35
80%	TRUE	103.83 ± 0.44	89.82 ± 0.11	102.04 ± 1.05	100.23 ± 2.62	103.81 ± 0.48
	SOE	101.31 ± 1.10	91.78 ± 0.64	99.62 ± 0.62	98.16 ± 1.98	101.88 ± 2.89
	OE-RR	103.72 ± 0.75	90.05 ± 2.49	100.87 ± 0.27	99.84 ± 1.09	104.36 ± 1.05
50%	TRUE	105.00 ± 0.35	95.69 ± 0.04	103.35 ± 1.01	102.50 ± 1.76	104.99 ± 0.36
	SOE	105.50 ± 2.96	102.05 ± 0.78	104.77 ± 2.13	104.84 ± 1.50	107.06 ± 2.19
	OE-RR	105.44 ± 1.40	96.55 ± 2.07	104.01 ± 0.66	103.42 ± 0.73	105.95 ± 0.77

Values in the table represent the mean position (mm) ± standard deviation (mm) of three independent repeated simulations.

**Table 5:** The mean error of the peak, 80% and 50% falloff positions (mm) of PGs between the Monte Carlo simulation (true) and that calculated by SOE and OE-RR for three independent simulations, respectively.

PG emissions		^14^N + ^12^C + ^15^O + ^16^O	^14^N	^12^C	^15^O	^16^O
Peak	SOE	−4.06	−6.65	−5.46	−5.75	−4.40
	OE-RR	−1.90	−3.53	−2.67	−2.30	−1.12
80%	SOE	−2.52	1.95	−2.42	−2.07	−1.94
	OE-RR	−0.11	0.23	−1.17	−0.39	0.55
50%	SOE	0.50	6.36	1.42	2.33	2.06
	OE-RR	0.44	0.85	0.66	0.92	0.95

To further evaluate the performance of the OE-RR algorithm, we also studied the reconstructions for different count levels of proton. As shown in Table 6, protons from 10^8^ to 10^10^ were simulated. for ^14^N + ^12^C + ^15^O + ^16^O, ^12^C, ^15^O and ^16^O PG emissions, it’s observed that the reconstructions were similar when we changed the count levels of proton. For ^14^N PG emission, as the count level increases, the estimated peak positions obtained by OE-RR are closer to the true values. The results in Table 6 also indicate that ^12^C, ^15^O and ^16^O PG emissions reconstructed by OE-RR were more suited for in proton beam range monitoring due to their higher accuracy of reconstruction and less dependence on the numbers of incident protons. Since the falloff positions at 80% and 50% predicted by using different count levels of protons have an accuracy of less than 1.5 mm, it is proved that the reconstruction of OE-RR algorithm can be used to accurately predicted the 80% and 50% falloff positions for PG emissions induced by different count levels of protons.

**Table 6:** The PGs’ peak and falloff at 80% and 50% positions(mm) obtained by the Monte Carlo simulation (true), SOE without resolution recovery, OE-RR with three different numbers of proton.

PG emissions		^14^N + ^12^C + ^15^O + ^16^O	^14^N	^12^C	^15^O	^16^O
Peak	TRUE	99.97 ± 0.89	86.09 ± 0.08	100.24 ± 0.66	97.53 ± 3.01	101.89 ± 0.09
	1.0 × 10^10^ protons	99.53 ± 0.21	84.87 ± 1.12	100.67 ± 0.34	97.13 ± 1.36	101.70 ± 0.01
	2.0 × 10^9^ protons	100.37 ± 0.63	84.73 ± 3.73	100.74 ± 0.27	98.66 ± 2.34	102.32 ± 0.32
	1.0 × 10^9^ protons	98.07 ± 0.90	82.56 ± 3.91	97.57 ± 0.51	95.22 ± 1.57	100.77 ± 1.35
	5.0 × 10^8^ protons	98.44 ± 1.43	79.84 ± 4.20	98.15 ± 0.97	95.42 ± 3.34	100.24 ± 1.07
	1.0 × 10^8^ protons	97.90 ± 1.07	75.80 ± 4.18	97.23 ± 0.64	95.03 ± 1.72	100.10 ± 1.34
80%	TRUE	103.83 ± 0.44	89.82 ± 0.11	102.04 ± 1.05	100.23 ± 2.62	103.81 ± 0.48
	1.0 × 10^10^ protons	103.59 ± 0.74	88.85 ± 0.12	102.28 ± 0.34	100.87 ± 1.35	102.82 ± 0.19
	2.0 × 10^9^ protons	103.87 ± 0.55	90.14 ± 0.19	102.47 ± 0.78	101.28 ± 1.98	103.92 ± 0.66
	1.0 × 10^9^ protons	103.72 ± 0.75	90.05 ± 2.49	100.87 ± 0.27	99.84 ± 1.09	104.36 ± 1.05
	5.0 × 10^8^ protons	102.70 ± 0.50	90.36 ± 1.15	100.93 ± 1.85	98.91 ± 1.80	103.78 ± 0.39
	1.0 × 10^8^ protons	102.65 ± 1.03	89.94 ± 1.93	100.73 ± 1.18	98.77 ± 1.49	103.10 ± 1.09
50%	TRUE	105.00 ± 0.35	95.69 ± 0.04	103.35 ± 1.01	102.50 ± 1.76	104.99 ± 0.36
	1.0 × 10^10^ protons	105.63 ± 0.59	96.41 ± 0.08	104.50 ± 0.30	101.95 ± 0.19	105.21 ± 0.11
	2.0 × 10^9^ protons	105.05 ± 0.46	96.04 ± 0.30	102.80 ± 0.53	103.28 ± 1.25	105.03 ± 0.45
	1.0 × 10^9^ protons	105.44 ± 1.40	96.55 ± 2.07	104.01 ± 0.66	103.42 ± 0.73	105.95 ± 0.77
	5.0 × 10^8^ protons	103.83 ± 0.49	96.53 ± 1.07	102.72 ± 1.24	101.80 ± 0.95	105.46 ± 0.93
	1.0 × 10^8^ protons	104.48 ± 0.85	94.80 ± 1.87	103.22 ± 1.28	101.90 ± 0.41	106.71 ± 0.89

Values in the table represent the mean position (mm) ± standard deviation (mm) of three independent repeated simulations.

### Reconstruction time

For this study, a 64-bit Linux computer, with a 2.50 GHz Intel i5-7200U CPU, was used to run the OE algorithm written in C++. For 100 times ordered OE iterations for 248 000 events (i.e. 24.8 million OE iterations), the reconstruction time for OE-RR was 28 s while the reconstruction time was 10 s for SOE algorithm. Both of them were utilized one thread with a single core.

## Discussion

In this paper, we studied the origin ensembles based reconstruction for PG imaging induced by proton pencil beam and detected by a three-stage CC in proton therapy from simulation. We made three modifications for OE-RR and investigated the improvement of each modification. Finally, we used the optimized OE-RR algorithm for PG reconstruction and evaluated its performance. Like previous studies^4,9^, the results of PG imaging show that the spatial resolution degradation caused by finite spatial and energy resolution of detection system reduces the accuracy of range verification based on reconstruction. The results also show that the optimized OE-RR algorithm realized better resolution recovery and more accurate estimation in range verification compared with SOE algorithm, as well as demonstrate the feasibility of this novel method in non-idealized PG-based proton range verification.

For ^14^N + ^12^C + ^15^O + ^16^O PG emissions, the OE-RR based reconstructions provided a better spatial resolution and more accurate estimation for the peak and falloff positions (i.e. 4.1 mm to 1.9 mm in Table 4) compared with the reconstructions obtained by SOE algorithm. Besides, the OE-RR based reconstructions can be used to predict the ^14^N + ^12^C + ^15^O + ^16^O PGs within 0.2 mm at 80% falloff positions and 0.5 mm at 50% falloff positions, respectively. Thus, it’s promising to monitor the beam range during proton therapy due to the accuracy of within 2 mm^4,12^.

As can be seen from the results, the PG produced by different kinds of atomic de-excitations (e.g. ^14^N, ^12^C, ^15^O, ^16^O) showed different correlations with the proton depth-dose curve, corresponding a previous study^13^. Thus, we also evaluated the reconstruction images of each characteristic, respectively. Our results show the OE-RR reconstruction can be used to predict the 80% and 50% falloff positions of ^12^C, ^15^O and ^16^O PG emissions within 1.2 mm or less, as well as predicted the peak position with an accuracy of less than 1.6 mm. Although the reconstructed ^14^N PG had a poor agreement with that obtained by Monte Carlo simulation at the peak position, the falloff at 80% and 50% positions were accurately predicted within 1.4 mm. According to one recent study^13^, the PGs derived from ^12^C and ^15^O de-excitation (i.e. 4.44 MeV and 5.25 MeV) have the similar distribution to the proton depth-dose curve, while the PGs derived from ^16^O de-excitation (i.e. 6.13 MeV) have the closest falloff correlation with the dose deposition curve in proton therapy. Therefore, the reconstruction of PG using OE-RR is able to accurately monitor *in vivo* dose deposition in proton therapy.

For the reconstruction of multi-energy PGs (e.g. ^14^N + ^12^C + ^15^O + ^16^O), the reconstruction process of the algorithm is the same as that of a single energy. However, the reconstructed images of multi-energy had a lower agreement with the Monte Carlo distribution, compared to that using single characteristic peak energy window (e.g. ^12^C, ^15^O or ^16^O). Since the ^14^N PG distribution had a lower agreement with dose deposition of proton beam compared with that of ^12^C and ^15^O^13^, the reconstruction contained ^14^N PG degraded the image quality and the accuracy of the image. But the good agreement of 80% and 50% falloff positions (within 1.4 mm) demonstrates the feasibility of this optimized OE-RR for multi-energy events, as well as the multiple interactions in detection system(e.g. triple events).

The reconstruction time for OE-RR is more than that of SOE algorithm due to the correction operation for deposited energy and intersected positions in per iteration (e.g. *N*_1_ × 11 times correction for *N*_1_ triple events, and *N*_2_ × 8 times correction for *N*_2_ double events, respectively). However, the speed of OE-based reconstruction was more than five times faster than PSF-based algorithm^10^, and the quality of reconstruction has good agreement shown above. Moreover, the reconstruction of OE-RR could further speed up by using parallel computing based on graphics processing unit (GPU), enabling the OE-RR algorithm to realize real-time image of PG.

Our results also indicated the requirement of the proposed method in the non-idealized system. For the large phantom used in our simulation, which is closer to the clinical application, the Compton camera (CC) is suggested to have the spatial resolution of less than 1 mm (i.e. the largest size of pixels is less than 2 mm × 2 mm), as well as the high energies resolution (i.e. semiconductor detectors like CZT or high purity germanium is more suitable due to their measurement error of deposited energies is within 1% for PGs). For the small phantom which can be detected with less distance between the PGs and detectors (i.e. nearly at 15 cm), the spatial resolution equals 2 mm of CC is sufficient for the accurate estimation in range verification. However, in both cases, the energy resolution of CC is suggested to within 1% for accurate reconstructions. Since the CZT detectors can work at room temperature without coolant, the size of the detection device can be less in volume and easier to move during treatment compared with that made by scintillation detector. The most complicated part in CC imaging is the processing of electronics and the records of coincident events. But we believe the problem can be solved well in real-world according the experiments implemented by Polf, J. C *et al*.^4^.

As for the imaging geometry or setup, since the reconstruction time of OE-RR is dependent on the number of detected events but there is almost no relation between the geometrical complexity of the imaging space (i.e. volume of interest) and the reconstruction time, the proposed method is promising to realize the real-time 3D reconstructions regardless of the choice about the size or bins. Besides, since the computing burden of OE reconstructions is only dependent on the number of events and generation of random numbers, the requirement of the CPU or storage capacity of imaging equipment is easy to meet.

The difficulties for different treatment sites are generally dependent on the distance between the CC (i.e. the first scattering detector) and the treatment site for the proposed method. The detection efficiency decreases as the distance increases, while the imaging quality also decreases as the distance increase due to the spatial degraded performance of cone intersection in CC imaging. Thus, the distance within 25 cm in real-world monitoring should be better. For the monitoring at longer distance, higher spatial (less than 1 mm) and energy resolution (less than 1%) of detectors may be required.

## Materials and Method

### Simulation and Detection of the PGs

The PGs emission and detection system were simulated by using Gate version 8.0 and Geant4 version 10.03 with QGSP_INCLXX physics list. The physics list QGSP_INCLXX, an experimental list, uses the Liege model to describe the inelastic interaction of protons and neutrons and it is suitable for simulating proton-induced interaction^14^. The standard physics option 3 was used to model the physical processes^15^. In the simulation, a 120 MeV proton pencil beam with a two-dimensional Gaussian spatial profile (σ = 3 mm~5 mm) was used, irradiating a large box water phantom (20 cm length × 20 cm width × 20 cm Depth) or a small water phantom (10 cm length × 10 cm width × 20 cm Depth). From 10^8^ to 10^10^ protons were simulated^16^.

**Figure 11:**
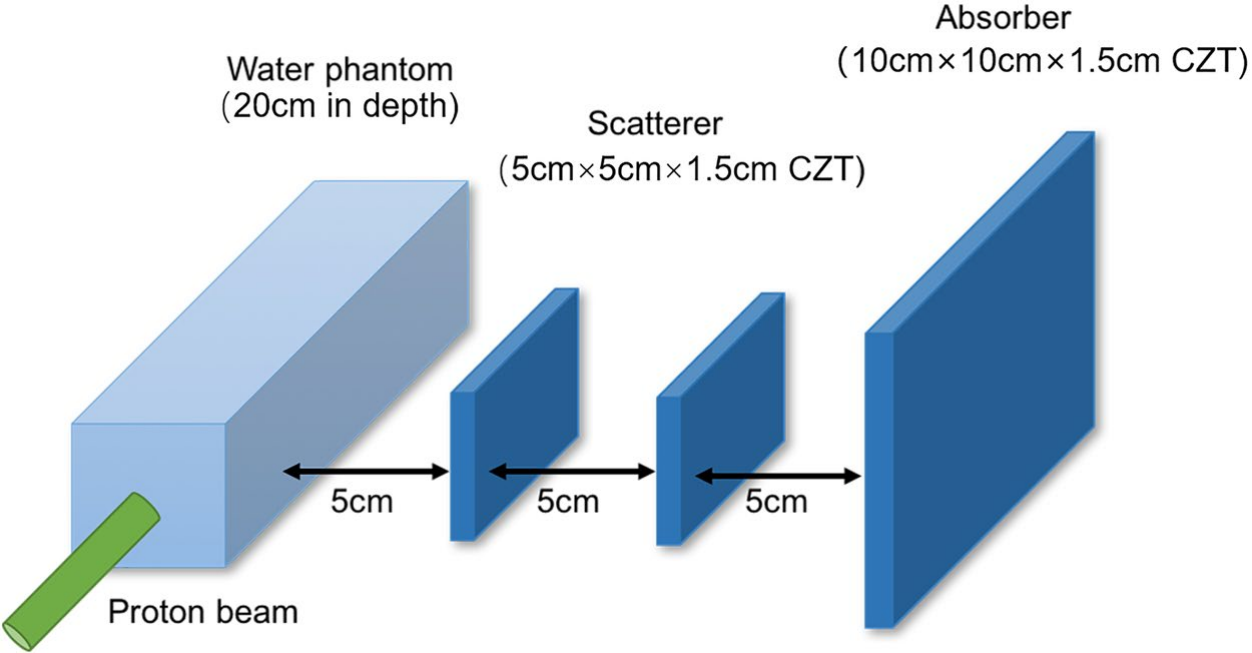
Simulation of the PGs produced by a proton pencil beam and the CC.

To detect PGs, we use a three-stage CC, consisting of three separate detection stages, and each is composed of several pixelated Cadmium Zinc Telluride (CZT) crystals. For this study, CZT was chosen for its high interaction cross section for gamma rays in our range of interest (up to ~7 MeV), as well as its high spatial and energy resolution, and the ability to record the depth information of interaction position by the pulse height and shape analysis of the anode signal^4^. Moreover, the three-stage structure has higher detection efficiency compared to two-stage for PG, because it can record both double-events (one scatter in the first detector and then are absorbed by the second detector) and triple-events (two times scatter in two different detectors and third interact in the other detector). For the detection the PGs from the small phantom, the first two stages contain a 25 × 25 array of pixelated CZT crystals (2 mm × 2 mm × 15 mm each) for a total CZT area of 5 cm × 5 cm, and the third stage contains a 50 × 50 array with the same pixelated CZT crystals for a total CZT area of 10 cm × 10 cm (As shown in Fig. 11). For the detection the PGs from the large phantom with the same total area of CZT, the pixelated CZT crystals were both set as 1 mm × 1 mm × 15 mm each due to the higher spatial resolution requirement of detectors for the farther detection distance. Adjacent stages ware separated from 5 cm. It was assumed that both detectors had a resolution of 1 mm in landscape and 1 mm in the depth direction. The CZT detectors’ energy resolution, *δ*_*CZT*_ (FWHM) is obtained by^17^:

1 $${\delta }_{CZT}={\rm{a}}+{\rm{b}}\sqrt{E}=2.355{\sigma }_{E}$$

where *E* is the deposited energy, *σ*_*E*_ is the standard deviation of spectral peak. For this study, both diploid events and triple events were recorded. The a and b parameters used in our simulation are −0.2892 and 0.4332, respectively. Both the parameters Referred to CZT pixel array detectors produced by IMDETEK Corporation Ltd, whose energy resolution is 5.13% for ^241^Am (59.5 keV) and 1.64% for ^137^Cs (662 keV)^17^. We performed an energy cut to only detect PGs with a total energy (determined using the Compton energy formulas^2,16^ in the range above 1 MeV to reject 511 keV annihilation gammas and most of background radiation. The simulation results of energy deposited of proton and PG are shown below.

**Figure 12:**
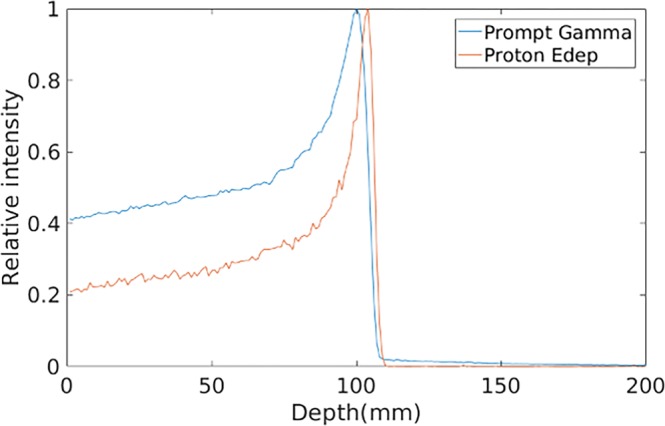
Energy deposited and PG distribution.

**Figure 13:**
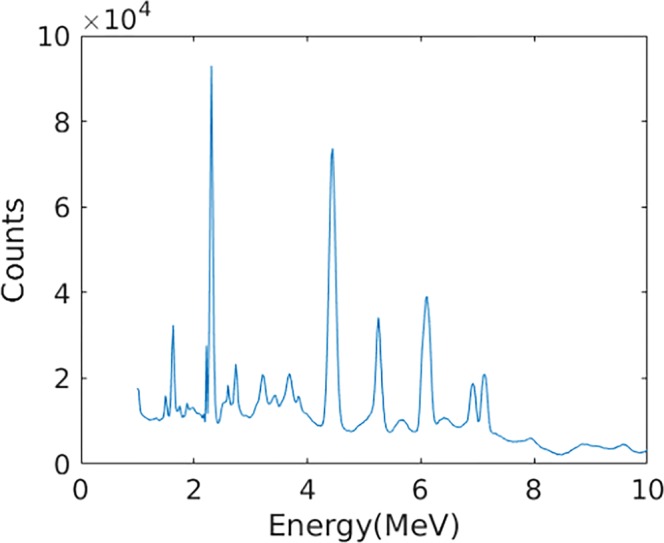
Energy spectrum of PG below 10 MeV.

As shown in Fig. 12, the relative intensity of PG decreases rapidly at the end of proton range and presents a close correspondence with the distal fall-off of BP, proving that the simulation results are reasonable. As shown in Fig. 13, four main PG energy spectral peaks are: 2.31 MeV (^14^N de-excitation), 4.44 MeV (^12^C de-excitation), 5.25 MeV (^15^O de-excitation) and 6.13 MeV (^16^O de-excitation)^18^. Since PG derived from ^14^N, ^12^C, ^15^O and ^16^O de-excitation exhibits different correlations with proton energy deposited in depth^13^, we evaluated the reconstruction by them respectively. The total energy windows of coincidence events were set as within ±0.2 MeV of the four known PG energy spectral peaks, to alleviate the affect due to the incomplete absorption and background radiation. For our designed Compton camera, the efficiency was 4.1 × 10^−4^ (*E*_*total*_ > 1 *MeV*) events per incident proton.

### Optimized OE-RR algorithm

The SOE algorithm is a Monte Carlo Markov chain method which uses the Metropolis–Hastings algorithm^19^. Rather than back-project the entire cone defined by detected gammas in the reconstruction, the SOE algorithm reproduces the image by considering only a single representative point on the conical surface. The representative points are initially chosen by randomly selecting a point on the conical surface and within the phantom volume. Each iteration of the algorithm attempts to improve the reconstruction by exchanging the current representative points with points where the probability of a gamma originating is higher. The reconstruction time using SOE approach is dependent on the number of detected events but there is almost no relation between the geometrical complexity of the region of response and the reconstruction time. Thus, SOE has a wide horizon of applications in emission tomography. The SOE algorithm has been used for PET, SPECT and CC image reconstruction^11,20^. For Compton-based PG images, SOE algorithm performed well with ideal CC^2^, but it couldn’t reproduce the PG distribution with a real-world CC because of the finite spatial and energy resolution^4^.

In the ideal case, the half conical surface corresponding to each CC event used in the OE reconstruction is defined by the exact deposited energies and the exact locations of interactions in the CC detectors. In fact, the conical surface, which is created using the measured detectors’ outputs, may not include the true event origin due to the limitations of the detection systems mentioned above, creating the deterioration of the spatial resolution in the reconstructed image.

In order to correct the finite energy and spatial resolutions, rather than using the output data of the measured detector to create the cones and implement the SOE iteration, OE-RR algorithm obtained the representative points on the “guess” cones determined by randomly corrected data corresponding to the measured data, which samples from the distributions of the positions of interactions in projection elements and deposited energies. After many iterations similar to SOE using these imaginary conical surfaces, the distribution of the sources will eventually be reconstructed.

In this study, we propose three modifications of OE-RR algorithm to improve its performance for PG image, and compare performance with the algorithm as described by Andreyev *et al*.^11^.

The first difference is that we referred to the modification of SOE in Mackin *et al*.^2^, stepped through the detected gammas one after another and calculated the acceptance probabilities A(Y_s_ → Y_s+1_) to implement ensemble transitions, where Y_s_ and Y_s+1_ are two subsequent, whereas Andreyev *et al*.^8,11^ select a detected gamma at random. Stepping sequentially through the list of detected gammas and ensuring that each gamma is tested once for each iteration improves the agreement between the MC true origin distribution and the SOE reconstructed origin distribution^2^.

The second difference is that we used random sampling method in sequence to obtain the initial event density distribution in the volume of interest (VOI) based on corrected values, whereas Andreyev *et al*.^8,11^ used the measured value. The sampling was uniform on the conical surface (which was in VOI at the same time) defined by obtaining samples from the distribution of the locations of interaction in projection elements and deposited energies. We evenly sampled *k* (*k* ≥ 1) points in VOI for each event. Since the true value of the interacted position and deposited energy is different from the measured value, using the corrected value (or called “guess” value) to generate the initial distribution is closer to the equilibration region, making the algorithm arriving faster to the equilibrium. Moreover, the sampling process can be implemented in parallel by dividing the event into several subsets. Thus, we can obtain a reasonable initial event density distribution in VOI with little time, while the convergence to equilibrium of the algorithm is remarkably faster.

The third difference is the correction for finite energy resolution. Since the PGs’ characteristic is different from the radioisotopes used in medical imaging, as well as the different detection methods, the correction method used in this study was different from that mentioned in Andreyev *et al*.^8^, aiming at improving the performance of OE-RR in PG image.

### Correction for finite energy resolution

In order to be able to “guess” the true deposited energy based on the measured energy, the posterior distribution of the true deposited energy is used. Different from the energies of photons emitted by the radioisotopes used in medical imaging, the exact energies of proton-induced PG are always unknown because of the prompt gammas’ secondary interaction in phantom. Besides, the detected events used for reconstruction contains triple events. The energy correction is implemented on the individual interactions stemming from a single source photon. The uncertainty of deposited energies in different stage were assumed to be independent. Thus, the corrected deposited energies used in our study is given as

2 $${E^{\prime} }_{i} \sim N({E}_{i},{\sigma }_{{E}_{i}}),\,with\,i=1,\,2$$

where *E*_*i*_ is the energy that is actually measured, *i* is the number of stage, ${\sigma }_{{E}_{i}}$ is the statistical standard deviation of *E*_*i*_, *N* is normal distribution defined by *E*_*i*_ and ${\sigma }_{{E}_{i}}$. For the CZT simulated in our study, ${\sigma }_{{E}_{i}}$ was given as

3 $${\sigma }_{{E}_{i}}=\eta \cdot \sqrt{{E}_{i}},\,with\,i=1,\,2$$

where *η* is the coefficient of proportionality between the sigma and the deposited energy is known^8^ (in our case 1.5%). In fact, the likelihoods ${\rm{p}}(\overline{{E}_{i}}|{E}_{i})$ has Gaussian shapes centered around $\overline{{E}_{i}}$ and standard deviations ${\sigma }_{\overline{{E}_{i}}}$. where the measurement *E*_*i*_ is assumed to be derived from the Gaussian distribution with true deposited energy $\overline{{E}_{i}}$ and standard deviation ${\sigma }_{\overline{{E}_{i}}}$. We assume that ${\sigma }_{{E}_{i}}\approx {\sigma }_{\overline{{E}_{i}}}$, then we can obtain a reasonable “guess” of ${E^{\prime} }_{i}$ by using (2), since the true deposited energy $\overline{{E}_{i}}$ could be derived form $N({E}_{i},\,{\sigma }_{{E}_{i}})$ in the case that *E*_*i*_ is the true deposited energy. The corrected deposited energies defined by (2) is used in each Markov step of the OE algorithm to determine the cone angle.

### Correction for finite spatial resolution

**Figure 14:**
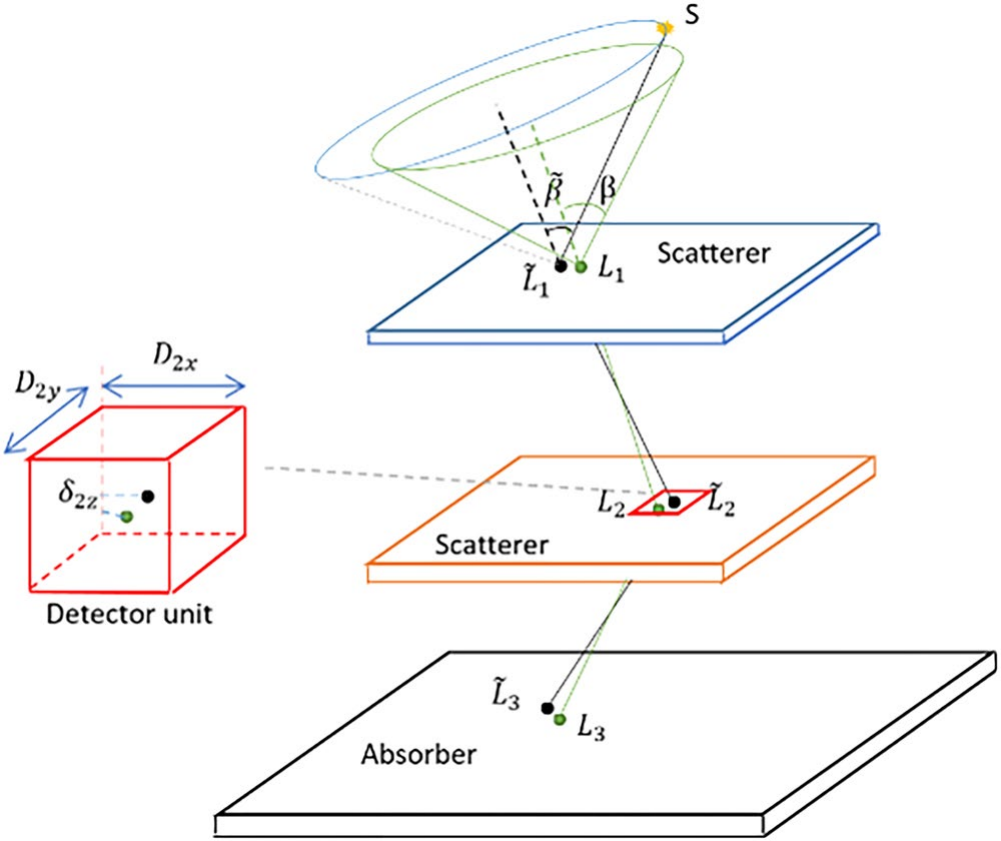
Compton camera acquisition principle (Take a triple event as an example). The true intersection positions of the photon emitted at S are ${\tilde{L}}_{1},{\tilde{L}}_{2},\,{\tilde{L}}_{3}$, and the deposited energies are ${\tilde{E}}_{1},\,{\tilde{E}}_{2},\,{\tilde{E}}_{3}$ for the three stages, respectively. The real scattering angle is $\tilde{\beta }$. The parameters measured by the camera are the locations *L*_1_, *L*_2_, *L*_3_, and the energies *E*_1_, *E*_1_ and *E*_3_, from which the Compton angle β is calculated. The point S where the photon is emitted lies on the cone having the apex in ${\tilde{L}}_{1}$, the axis collinear with the scattered ray direction and the half-opening angle $\tilde{\beta }$.

For the correction of spatial resolution, we modify the detection data based on the size of the detection unit in x-y plane and the measure error in z (depth) direction as shown in Fig. 14. The data measured by the detector is ${L}_{i}=({\bar{x}}_{i},{\bar{y}}_{i},{z}_{i})$, where the $({\bar{x}}_{i},{\bar{y}}_{i})$ is the central coordinate of the interacted detection unit (projection element) in x-y plane. For the case that the scale of the detection unit is much smaller than the detection distance, it can be considered that the actual intersection location is uniformly distributed in the detection unit. Assuming that the spatial scale of the *i*-th detection unit is *D*_*i*_, the correction value ${L^{\prime} }_{i}=({x^{\prime} }_{i},{y^{\prime} }_{i},\,{z^{\prime} }_{i})$ is given by,

4 $${x^{\prime} }_{i} \sim U({x}_{i}-\frac{{D}_{ix}}{2},\,{x}_{i}+\frac{{D}_{ix}}{2})$$

5 $${y^{\prime} }_{i} \sim U({y}_{i}-\frac{{D}_{iy}}{2},\,{y}_{i}+\frac{{D}_{iy}}{2})$$

6 $${z^{\prime} }_{i} \sim U({z}_{i}-\frac{{\delta }_{iz}}{2},\,{z}_{i}+\frac{{\delta }_{iz}}{2})$$

where *U* is the uniform distribution, *δ*_*iz*_ is the measure error in the *z* direction for *i*-th stage.

When an event is considered by the OE algorithm, a new position of interaction within the detector unit is selected according to the distribution defined by (4) ~ (6). Unlike the correction for finite energy resolution where two random number was generated to determine a sample from the normal distribution, the implementation of the correction for spatial resolution requires a generation of six random numbers for double events, night random numbers for triple events, respectively. Three random numbers are needed to simulate the detection uncertainty in the x, y, and z directions of the detector elements for each of the detectors.

### Reconstruction using OE-RR

The modifications and corrections described above were combined in the optimized OE-RR algorithm to allow for the full modeling of the Compton camera resolution due to limited detectors’ resolutions. Taking the detected triple event as an example and assuming the information for each detected event that we measured is (*L*_1_, *L*_2_, *L*_3_, *E*_1_, *E*_2_, *E*_3_), in the OE-RR reconstruction the following steps were executed:

1. For each detected event, the identifier was randomly defined when input and store the list-mode data. We used the random sampling method in sequence to obtain the initial event density distribution in the VOI based on the corrected values $({L^{\prime} }_{1},{L^{\prime} }_{2},{L^{\prime} }_{3},{E^{\prime} }_{1},{E^{\prime} }_{2},{E^{\prime} }_{3})$. The sampling was uniform on the conical surface (which was in VOI at the same time) defined by obtaining samples from the distribution of the positions of interaction in projection elements and deposited energies defined by (2~6). We evenly sampled k (k ≥ 1) points in VOI for each event.
2. Event *n*_*l*_ (*l is the identifier*, *from* 1 *to N*, *where N is the total number of detected events*) was chosen and voxel *i* within VOI that contained the origin of this event was recorded.
3. The parameters of the new half cone corresponding to event *n*_*l*_ were stochastically determined by obtaining samples from the distribution defined by (2~6).
4. Event *n*_*l*_ was randomly moved to a new position *j* on the half-cone surface determined in step 4 within VOI and voxel *j* which contained this new position was recorded.
5. A random number was generated. The new location of the event *n*_*l*_ was accepted if this random number (from a range [0, 1]) was smaller than (c_*j*_ + 1) ϵ_*i*_/c_*i*_ϵ_*j*_ where c_j_ and c_i_ were the numbers of event origins contained in voxels *j* and *i* before the move and ϵ_*i*_ and ϵ _*j*_ were these voxel sensitivities.
6. Set *l* = *l* + 1, and repeated steps 2 to 5. *N* times repetition corresponds to one iteration of this OE-RR.
7. Repeated step 6 until reaching the iterations of this OE-RR algorithm.

In our case, the voxel sensitivity was assumed uniform in VOI. The above algorithm reaches equilibrium when on average the number of events in each voxel remains constant i.e., events move in and out the voxels in Markov moves, but on average the number of event origins in each voxel remains the same. Finally, the weighted number of events in each voxel determined the probability distribution of sources in VOI.

## Conclusion

The optimized OE-RR proposed in this paper leads to a good resolution recovery and could be used to predict the falloff at 80% and 50% positions of PG emissions in proton therapy with an accuracy of less than 1.6 mm. Moreover, the time consumption for OE-based reconstruction of PG is less than 1 minutes, which can be further speed up by using parallel computing. Thus, this approach is feasible to realize the high accuracy and real-time *in vivo* verification based on prompt gamma imaging in proton therapy.
